# 

**Published:** 1882-04

**Authors:** 


HERMAN MYNTER, M. D.

### CONTENTS:

PAGE.

ORIGINAL COMMUNICATIONS:

The Deliverances of the Retina. By F. W. Abbott, M. D., . . . . . . 385
Oxygen and Protoplasm. By W. H. Pitt, M. D., . . . . . . . . . . 396
Self-Adjusting, Shell-Plate Entropium Forceps. By Dr. Rœhrig, Cornell University, Ithaca, N. Y., . . . . . 401

CLINICAL REPORTS:

Strangulated Umbilical Hernia—Herniotomy—Cure. Reported by Herman Mynter, M. D., . . . . . . . . 402

SELECTIONS:

The Treatment of Typhoid Fever, . . 404

CORRESPONDENCE:

Letter from Vienna. By Frederick Peterson. . . . . . . . . . 418

EDITORIAL:

The American Medical Profession, . . 427
William Gould, M. D. (Obituary). . . 429
Editorial Notes. . . . . . . . 430

REVIEWS, . . . . . . . 432

